# A case report of pediatric neurotrophic keratopathy in pontine tegmental cap dysplasia treated with cenegermin eye drops

**DOI:** 10.1097/MD.0000000000020816

**Published:** 2020-07-24

**Authors:** Riccardo Fausto, Roberto Ceccuzzi, Eleonora Micheletti, Riccardo Clerici, Ivano Riva, Andreas Katsanos, Francesco Oddone, Luciano Quaranta

**Affiliations:** a Department of Surgical, Clinical, Diagnostic and Pediatric Sciences, Section of Ophthalmology, University of Pavia - IRCCS Fondazione Policlinico San Matteo; b IRCCS Fondazione G.B. Bietti, Rome, Italy; c Department of Ophthalmology, University of Ioannina, Ioannina, Greece.

**Keywords:** congenital neurotrophic keratopathy, eye drops, persistent epithelial defect, pontine tegmental cap dysplasia, recombinant human nerve growth factor

## Abstract

**Rationale:**

To report the management of recalcitrant neurotrophic keratopathy in a pediatric patient affected by pontine tegmental cap dysplasia (PTCD) using topical human recombinant nerve growth factor (hrNGF, Cenegermin 20 μg/ml). To the best of our knowledge the present case is one of the few described in patients with congenital NK treated with Cenegermin, and the first in a patient affected by PTCD.

**Patient Concerns:**

A 9-year-old patient, affected by PTCD with bilateral cranial nerve V_1_ and VIII palsies, was referred to our hospital for visual disturbances and redness of the right eye due to persistent neurotrophic epithelial defect. The patient presented marked developmental delay, ataxia, bilateral hypoacusia, and bilateral corneal severe hypoaesthesia. Ocular history revealed multiple treatments in order to treat neurotrophic ulcer in the left eye. Four years later, he developed a persistent epithelial defect with corneal anesthesia in the right eye.

**Diagnoses:**

The impaired trigeminal nerve function, due to the underlying congenital disease, led to the development of moderate NK (stage II) in the right eye and a mild NK (stage I) in the left eye.

**Interventions:**

Cenegermin 20 μg/ml eye-drop was administered in both eyes. Treatment was continued for 8 weeks. The patient was assessed after 4 and 8 week of treatment. At each follow-up visit, treatment efficacy and adverse events were evaluated.

**Outcomes:**

The use of Cenegermin eye drops facilitated the remarkable resolution of the neurotrophic keratopathy and the improvement of corneal sensitivity in both eyes. No local or systemic adverse events were observed.

**Lessons:**

Topical Cenegermin 20 μg/ml was well-tolerated and may represent a valuable therapeutic option in the management of pediatric neurotrophic keratopathy.

## 1 Introduction

Neurotrophic keratopathy (NK) refers to a condition in which damage at any level of the trigeminal nerve leads to corneal sensitivity impairment accompanied by nonhealing epithelial defects with or without stromal ulceration.^[1–3]^ Several systemic and ocular conditions can lead to NK. Most common causes of severe NK are herpetic keratitis, ocular chemical burns, contact lens abuse and neurosurgical procedures.^[1,4]^ Among uncommon causes that can lead to NK, congenital diseases such as familial dysautonomia (Riley-Day syndrome), Goldenhar-Gorlin syndrome, Mobius syndrome, and familial trigeminal anesthesia have also been described.^[2,5]^

Topical therapies such as artificial tears, serum/plasma eyedrops, anti-inflammatory and antibiotic agents are commonly used.^[1]^ Further therapeutic strategies include contact lenses’ use, amniotic membrane transplantation, conjunctival flap transposition, tarsorrhaphy, and corneal surgery.^[4,6,7]^ Human recombinant nerve growth factor (hrNGF) has been recently approved as the specific topical treatment for NK because of its potential to target the underlying nerve deficit.^[3,8,9]^

To the best of our knowledge, only few cases of patients with congenital NK treated with Cenegermin have been reported.^[2,5]^

We report the use of hrNGF (Cenegermin 20 μg/mL eye drops—Oxervate, Dompè Farmaceutici S.p.A, L’Aquila, Italy) in a pediatric patient with Pontine Tegmental Cap Dysplasia (PTCD) associated with NK due to trigeminal nerve dysfunction.

## 2 Case report

A 9-year-old boy with PTCD has been managed in our institution. PTCD is a rare brainstem malformation characterized by the pontine tegmentum projecting into the fourth ventricle, ventral pontine, and cerebellar vermal hypoplasia.^[10]^ To the best of our knowledge, <50 cases of PTCD have been reported in the literature so far.^[11]^ Patients with this condition suffer from several cranial and extracranial abnormalities, pyramidal and cerebellar malformations, and various cranial nerves dysfunctions (VIII cranial nerve is always involved, whereas V, VI and VII cranial nerves are variably involved). The main symptoms and signs include auditory deficits, cognitive impairment, ataxia, deglutition dysfunction, abnormalities of eye movements, variable deficiency of the trigeminal nerve, and other neurological dysfunctions. The treatment and prognosis depend on the range and severity of symptoms and aim to improve the quality of life of these patients.^[10]^

The patient was referred to our hospital for the first time in January 2014 for visual disturbances and redness of the left eye. He suffered from marked developmental delay, ataxia, bilateral hypoacusia, and bilateral corneal severe hypoaesthesia. At this visit, eye movements were normal. Facial expression was also normal and eyelid closure during sleep had been satisfactory. Visual acuity could not be determined due to the patient's mental state and lack of cooperation. Slit-lamp examination revealed a corneal epithelial defect with fluorescein staining in the right eye, and a corneal ulcer with stromal melting in the left eye. A cotton wisp that was used to qualitatively assess corneal sensitivity showed no blinking response in any eye. Cochet-Bonnet esthesiometry was impossible to perform due to the patient's lack of cooperation. Amniotic membrane transplantation was performed in the left eye 5 days after the first examination. The procedure did not prove effective in preventing corneal melting, and 1 month later a deep anterior lamellar keratoplasty (4 mm in diameter) was performed along with lateral marginal tarsorrhaphy in the left eye. Four months later, the corneal ulcer had healed.

Over the next 4 years, both eyes were being treated with preservative-free artificial tears and antibiotic ointment for the occasional epithelial defects.

**Figure 1:**
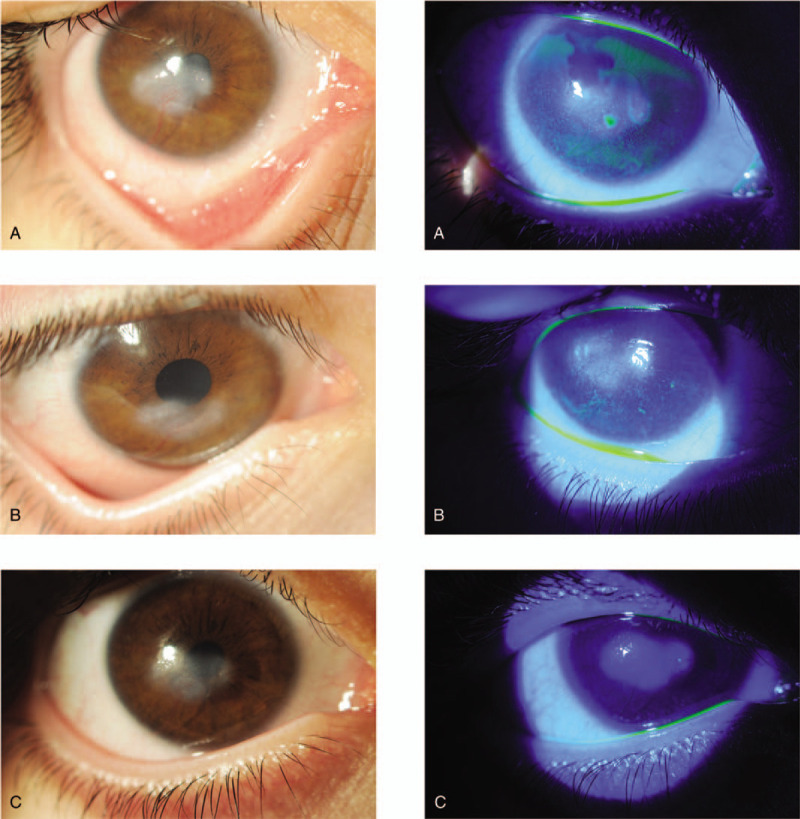
(A) Right eye at baseline before starting the treatment with Cenegermin 20 μL/mL. (B) Right eye at week 8 (end of the treatment). (C) Right eye at month 6 from baseline.

In December 2018, the patient was evaluated during a routine follow-up visit. Slit lamp examination revealed a central corneal opacity with stromal neovascularization, and corneal epithelial defect associated with corneal anesthesia in the right eye. The left eye exhibited diffuse punctate epithelial erosions with corneal hypoesthesia. The patient did not complain of any symptoms. No signs of anterior chamber inflammation or infection were observed (Fig. 1A).

Nonhealing neurotrophic epithelial defect (classified as NK stage II or moderate NK)^[1]^ in the right eye and a mild NK (stage I) in the left eye was diagnosed. Considering that for >4 years our patient has undergone medical and surgical interventions without a long lasting therapeutic effect, we decided to commence treatment with hrNGF which had become available at the time.

In Italy, HrNGF is not approved for use in pediatric patients (off-label), whereas it is approved in the United States. In consideration of the patient's clinical presentation and supporting clinical data, Italian Medicines Agency approved and supported the use of hrNGF.

HrNGF was administered after written informed consent was obtained from the guardians of the patient to be treated.

Cenegermin 20 μg/mL was administered in the right eye for 8 weeks as follows: 1 drop in the conjunctival sac 6 times a day at 2 hourly intervals, from 8 am to 8 pm. The patient was assessed 4 and 8 weeks after the treatment was started. At each follow-up visit, slit-lamp examination and esthesiometry were performed. In vivo confocal microscopy for the assessment of possible corneal nerve changes and regeneration was not performed due to the patient's poor cooperation.

At week 4, the corneal opacity was slightly reduced, and smoothness of the corneal epithelium was improved. Corneal sensitivity was assessed with a cotton wisp and was deemed improved. Some authors reported a potential vascular regulatory function of NGF. In particular, in a mouse model, murine-NGF seems to regulate corneal neovascularization modifying stromal blood flow and supporting innervation of perivascular nerves.^[12]^ After 8 weeks of treatment, the corneal neovascularization was markedly reduced, the opacity had significantly decreased and the epithelium seemed completely normal (Fig. 1B). Over the next 6 months, the patient's eye remained comfortable, the epithelium was normal, the opacity decreased in size and density, and only fine neovascularization had remained (Fig. 1C).

**Figure 2:**
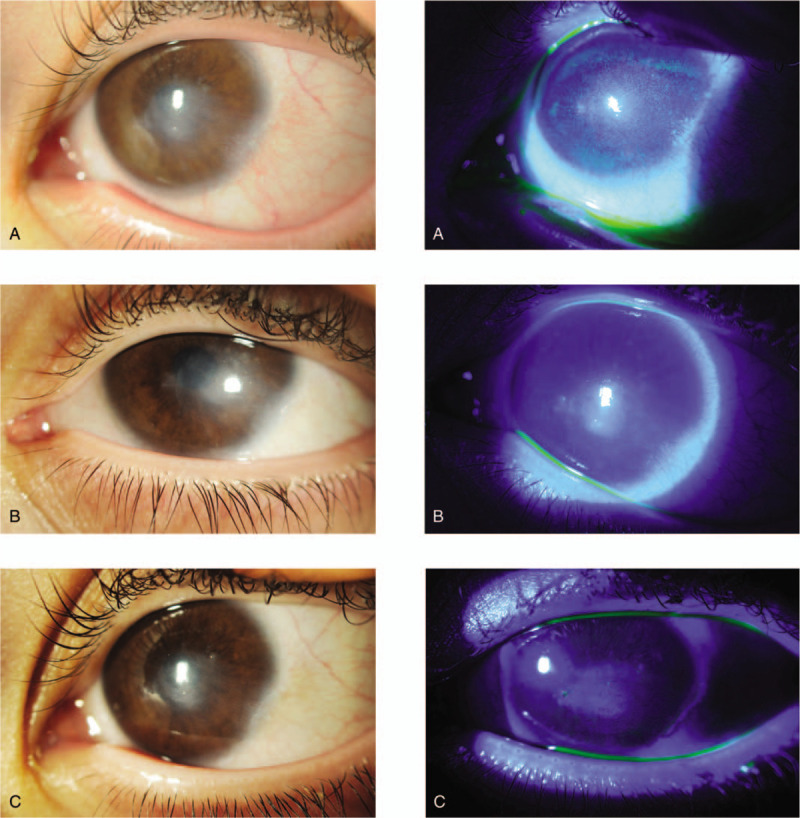
(A) Left eye at baseline. (B) Left eye at week 8 (at the end of the treatment with Cenegermin 20 μl/ml). (C) Left eye at 6 months after treatment.

The patient's left eye was also diagnosed with NK stage I; therefore, treatment was started also in the left eye. Resolution of the superficial punctate keratopathy and improvement in corneal sensitivity was noted over the course of the treatment (Fig. 2).

## 3 Discussion

PTCD is a rare congenital neurological syndrome characterized by ataxia, developmental delay, and multiple cranial nerves involvement. In the reported case, corneal hyposensitivity due to the impaired function of the trigeminal nerve appears to be responsible for NK. Corneal nerve involvement due to trigeminal dysfunction has been reported in 15 of the 25 described PTCD patients.^[13]^ When present, neurotrophic epithelial defects and ulcers have been treated with lubricating ointments, soft contact lenses, maternal serum eyedrops amniotic membrane transplantation, and other surgical approaches.^[6,7]^ However, these conditions are frequently associated with poor visual outcomes, and the management requires long-term efforts with multiple treatments, especially in pediatric patients.^[6]^

The usual approaches with artificial tears, autologous serum, antibiotic ointments, and contact lenses aim to prevent the progression of corneal damage and promote epithelial and stromal healing. Unfortunately, these modalities are frequently ineffective. Surgical procedures like tarsorrhaphy, conjunctival flaps, and amniotic membrane transplantation provide significant relief, but may cause visual and esthetic problems. This can be an important concern, particularly in pediatric patients. Although lamellar or full-thickness corneal graft procedures can be viable choices when stromal opacities involve the visual axis, such interventions have guarded prognosis in NK patients who suffer from impaired epithelial wound healing and dry eye.^[1]^

Management of NK has recently changed with the introduction of Cenegermin. This agent is a recombinant form of human nerve growth factor produced by *Escherichia coli* as a pro-peptide, which is then cleaved to its mature form. NGF is a neurotrophin vital for the development of sensory neurons, as well as their trophic support and survival following injury.^[3,8]^ It is involved along with regenerating agents (Coenzyme Q10, Cacicol 20, Thymosin beta 4, P substance) in proliferation and differentiation of epithelial cells, induction of stromal healing, and remodeling by hindering inflammation and supplying binding sites for growth factors in the corneal stroma.^[8,14]^

The safety and efficacy of hrNGF eye-drops (Cenegermin 20 μg/mL) has been successfully tested in phase I and II clinical trial in adult patients (n = 156) with moderate (stage II) and severe (stage III) NK neurotrophic keratopathy. Adverse events (AE) occurred in 25 patients and the most common are ocular pain, and redness, lachrymation, eyelid pain, and corneal neovascularization. Despite of the risk of developing these mild AEs, the efficacy of Cenegermin 20 μg/mL in stimulating complete corneal healing has been demonstrated in 70% of patients with moderate and severe NK.^[9]^

Moreover, topical hrNGF was recently evaluated in dry eye patients and found to be useful in increasing tear production and improving tear film stability.^[15]^ In the reported clinical case, we feel that the use of hrNGF facilitated the healing of the persistent epithelial defect, the resolution of the opacity, and the improvement of corneal sensitivity. We also believe that our treatment with hrNGF expedited the healing of the recalcitrant corneal surface problems in both eyes. Although no adverse events were observed in our pediatric patient with multiple comorbidities, more studies may be needed to assess the long-term safety profile of topical hrNGF in this age group.

To the best of our knowledge, this is first case of PTCD associated with congenital NK who was successfully treated with Cenegermin.

## Author contributions

**Conceptualization:** Riccardo Fausto, Roberto Ceccuzzi, Luciano Quaranta.

**Investigation:** Riccardo Fausto, Roberto Ceccuzzi, Riccardo Clerici, Eleonora Micheletti.

**Methodology:** Roberto Ceccuzzi.

**Supervision:** Luciano Quaranta, Ivano Riva.

**Visualization:** Ivano Riva, Riccardo Fausto.

**Writing – original draft:** Riccardo Fausto, Eleonora Micheletti.

**Writing – review & editing:** Luciano Quaranta, Roberto Ceccuzzi, Andreas Katsanos, Francesco Oddone.
